# Oesophageal cell collection device and biomarker testing to identify high-risk Barrett's patients requiring endoscopic investigation

**DOI:** 10.1093/bjs/znae117

**Published:** 2024-05-13

**Authors:** Siobhan Chien, Paul Glen, Ian Penman, Neil Cruickshank, Gavin Bryce, Andrew Crumley, Perminder Phull, Michael Miller, Jonathan Fletcher, Ivan Gunjaca, Jeyakumar Apollos, Kevin Robertson, Grant Fullarton

**Affiliations:** School of Cancer Sciences, University of Glasgow, Glasgow, UK; Centre for Sustainable Delivery, Golden Jubilee National Hospital, Glasgow, UK; Department of General Surgery, Queen Elizabeth University Hospital, Glasgow, UK; Centre for Liver & Digestive Disorders, Royal Infirmary of Edinburgh, Edinburgh, UK; Department of General Surgery, Victoria Hospital, Kirkcaldy, UK; Department of General Surgery, University Hospital Wishaw, Wishaw, UK; Department of General Surgery, Forth Valley Royal Hospital, Larbert, UK; Department of Gastroenterology, Aberdeen Royal Infirmary, Aberdeen, UK; Department of Gastroenterology, Ninewells Hospital, Dundee, UK; Department of Gastroenterology, Borders General Hospital, Melrose, UK; Department of Gastroenterology, Raigmore Hospital, Inverness, UK; Department of General Surgery, Dumfries & Galloway Royal Infirmary, Dumfries, UK; Department of General Surgery, University Hospital Crosshouse, Kilmarnock, UK; Centre for Sustainable Delivery, Golden Jubilee National Hospital, Glasgow, UK

## Abstract

**Background:**

Barrett's oesophagus surveillance places significant burden on endoscopy services yet is vital to detect early cancerous change. Oesophageal cell collection device (OCCD) testing was introduced across Scotland for Barrett's surveillance in response to the COVID-19 pandemic. This national pragmatic retrospective study presents the CytoSCOT programme results and evaluates whether OCCD testing is successfully identifying high-risk Barrett's patients requiring urgent endoscopy.

**Methods:**

All patients undergoing OCCD testing for Barrett's surveillance across 11 Scottish health boards over a 32-month period were identified. Patients who underwent endoscopy within 12 months of OCCD test were included. Individual patient records were interrogated to record clinical information and OCCD test result to categorize patients into risk groups. Endoscopic histopathology results were analysed according to risk group and segment length. Patients were deemed high risk if the OCCD test demonstrated atypia and/or p53 positivity.

**Results:**

4204 OCCD tests were performed in 3745 patients: 608 patients underwent endoscopy within 12 months and were included in this analysis. Patients with longer Barrett's segments were significantly more likely to have an abnormal OCCD test. 50/608 patients (8.2%) had high-grade dysplasia or cancer on endoscopic biopsies: this equates to 1.3% of the total group (50/3745). 46/50 patients (92.0%) were deemed high risk, triggering urgent endoscopy: this rose to 100% with insufficient tests removed. There were no cancers diagnosed within 12 months post-OCCD in the low-risk group.

**Conclusion:**

OCCD testing is an effective triage tool to identify high-risk patients with Barrett's oesophagus requiring further investigation with endoscopy within the real-world setting.

## Teaser

Oesophageal cell collection device (OCCD) testing has shown promising trial results in the diagnosis of Barrett's oesophagus. This study presents the first real-world data to show that OCCD testing demonstrates sound diagnostic accuracy as a triage tool in highlighting high-risk Barrett's surveillance patients requiring urgent upper gastrointestinal endoscopy.

## Introduction

Oesophageal adenocarcinoma (OAC) is associated with dismal 5-year survival rates of less than 25%^1^. Barrett's oesophagus is a well-established precursor lesion to OAC, in which columnar-lined epithelium demonstrating intestinal metaplasia (IM) replaces the usual squamous epithelial lining of the oesophagus. IM has the propensity to progress through low-grade dysplasia (LGD), high-grade dysplasia (HGD) and intramucosal cancer (IMC), before developing into invasive OAC^2^. Although the overall estimated annual risk of progression to OAC is low (0.3%)^3^, this increases exponentially to 16.3% when dysplasia is present^4^. High-quality endoscopic Barrett's surveillance programmes not only facilitate early detection of neoplastic change, but also enable therapeutic intervention in cases of dysplasia, thus improving OAC-related morbidity and mortality rates in the Barrett's population^5^.

Although upper gastrointestinal (UGI) endoscopy is the gold standard for Barrett's surveillance, sampling bias and user error can impact histopathology results. The rate of missed OAC within 1 year of index Barrett's surveillance endoscopy is estimated to be 23–30%^6–8^. Furthermore, Barrett's surveillance programmes demand substantial clinical and administrative resources. During the COVID-19 pandemic, all routine endoscopy was temporarily halted across NHS Scotland^9^. Clearly, there is a requirement for innovative technologies to enhance Barrett's surveillance programmes.

A minimally invasive, non-endoscopic test using an oesophageal cell collection device (OCCD) has been developed, allowing collection of pan-oesophageal mucosal cells for cytological analysis to detect the immunohistochemical biomarker trefoil factor 3 (TFF3)^10^. TFF3 is a marker of columnar epithelium, suggesting the presence of IM. Furthermore, the addition of an OCCD biomarker panel effectively detects dysplasia and neoplasia: the collected cells are tested for atypia (suggestive of inflammation or dysplasia) and p53 (the most prevalent biomarker for malignant transformation in Barrett's oesophagus)^11,12^. OCCD testing demonstrates over 92% specificity and 80% sensitivity in the diagnosis of Barrett's oesophagus: its specificity rises to 94% when insufficient samples are excluded^13^. Furthermore, OCCD testing is well tolerated by patients, with a failure to swallow rate of only 5.0%^14^.

OCCD testing was launched into clinical practice across Scotland in September 2020 as an emergency response to the COVID-19 pandemic. Fully funded by NHS Scotland, the CytoSCOT (ScottishCytologyOesophagealTest) programme saw OCCD testing rolled out at pace across all mainland Scottish health boards^15^. Although trial data have shown OCCD testing increases detection of Barrett's oesophagus 10 times over the current standard of care within the reflux screening population^10^, its role as a triage tool in Barrett's surveillance remains unclear. This study is the first to analyse the correlation between OCCD test result and subsequent endoscopic histopathology biopsies in clinical practice. The aim was to evaluate whether OCCD testing is successfully identifying high-risk Barrett's oesophagus patients requiring endoscopy in the real-world setting to validate the ongoing use of OCCD testing for Barrett's oesophagus in clinical practice.

## Materials and methods

Patients who underwent OCCD testing using Cytosponge™ for surveillance of Barrett's oesophagus between 14 September 2020 and 30 April 2023 were identified using prospectively maintained local databases from 11 Scottish health boards. Patients were recruited for OCCD testing if previously entered in local Barrett's surveillance programmes, where prior endoscopy demonstrated macroscopic changes consistent with Barrett's oesophagus (i.e. salmon-coloured mucosa progressing cephalad from the gastro-oesophageal junction). The presence of IM on endoscopic biopsies was not considered a prerequisite for entry into surveillance, as per the British Society of Gastroenterology (BSG) guidelines^2^. All patients who subsequently underwent UGI endoscopy within 12 months of OCCD test with available histopathology results were identified and included in this analysis. Forward referral for endoscopy was subject to clinicians’ discretion at local health board level.

All OCCD tests were processed centrally in a UK-based diagnostic laboratory (Cyted Ltd), as previously described^16^. Processed results from Cyted Ltd were returned to the requesting hospital, with ongoing management decided locally.

Individual patient records were interrogated, with baseline demographics, previous Barrett's endoscopic morphology and pathology, and OCCD test result recorded retrospectively to form the national CytoSCOT registry. Indication for UGI endoscopy, endoscopic biopsy results and subsequent clinical management were also documented. Barrett's segment length was determined using the Prague classification: short segment was defined as M < 3 cm; long segment was defined as M ≥ 3 cm to <10 cm; ultra-long segment was defined as M ≥ 10 cm. Patients were further subcategorized into ultra-low, low, medium and high-risk groups based on OCCD test results and clinical details as published by Landy *et al.*^17^ (*Fig. S1*).

The primary outcome was presence of HGD or cancer within endoscopic biopsies. The secondary outcome was diagnosis of any grade of dysplasia within the endoscopic biopsies, based on Barrett's risk group. Exclusion criteria included: OCCD test for reflux symptoms; patients who did not undergo UGI endoscopy within 12 months of OCCD test; outstanding histopathology results at the time of analysis; previous endoscopic treatment for dysplasia.

Patients were not actively involved in study design or analysis. Ethical approval was obtained for this study via information governance teams in each health board, in addition to national Public Benefit and Privacy Panel (PBPP) approval from the Caldicott guardian.

### Statistical analysis

Continuous parameters were presented as median and interquartile range and categorical data as counts and percentages. The chi-squared test was performed for comparison of categorical variables, where appropriate. *P* ≤ 0.05 was considered statistically significant. Statistical analysis was performed using SPSS software version 28.0 (SPSS Inc., Chicago, IL, USA).

## Results

5590 OCCD tests were performed across 11 Scottish health boards within the study period; 4204 OCCD tests (75.2%) were performed on 3745 patients for Barrett's surveillance: the additional 459 OCCD tests (10.9%) were repeat procedures for insufficient samples (i.e. insufficient glandular groups for analysis in the surveillance setting; *n* = 229) or clarification of initial results, where the first test showed TFF3 negativity (*n* = 230).

**Fig. 1:**
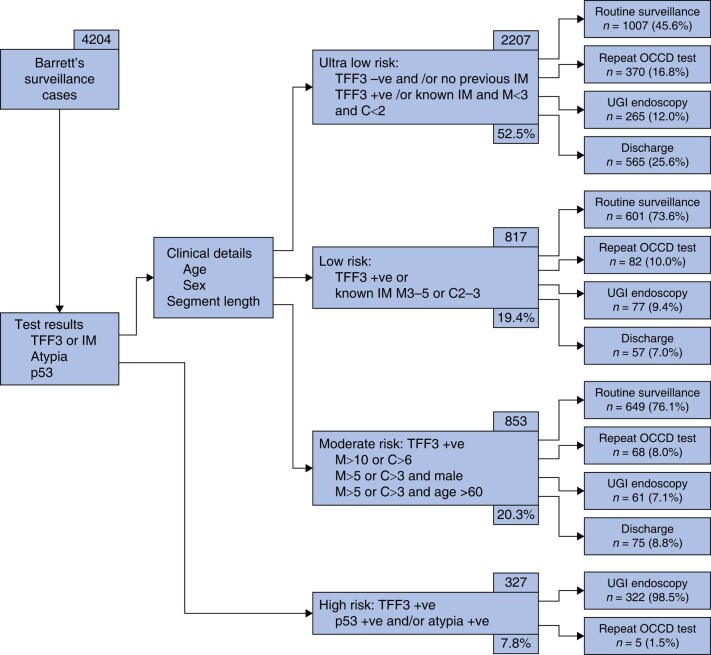
Patient workflow and clinical management within the Barrett's surveillance cohort, categorized by risk group (*n* = 4204)

*Figure 1* demonstrates patient workflow and initial clinical management, categorized by risk group. 322/327 high-risk patients (98.5%) were referred for urgent endoscopy. 697/3745 patients (18.6%) were discharged from Barrett's surveillance based on OCCD test result and clinical details: this included those within the ultra-low-risk group with 2 negative tests for IM or those >80 years of age. 75/853 patients (8.8%) were discharged within the moderate-risk group: in all cases, this was due to age and co-morbidity.

608/3745 patients (16.2%) underwent UGI endoscopy within 12 months of OCCD test and were included in this analysis (*Fig. S2*). The median age was 67 years (i.q.r. 60–73) and 427/608 patients (70.2%) were male. The median follow-up time was 14 months (i.q.r. 8–22). The median time from last surveillance endoscopy to OCCD test was 38 months (i.q.r. 29–48), with 509/608 patients (83.7%) demonstrating IM on previous endoscopic biopsies.

*Table 1* demonstrates OCCD biomarker result according to Barrett's segment length. Within the cohort, 124/608 tests (20.4%) were returned as insufficient: in the whole Barrett's cohort, 456/4204 tests (10.8%) were deemed insufficient for analysis (*Table S1*). 229/456 initial insufficient tests (50.2%) underwent repeat OCCD testing, 131 (28.7%) proceeded direct to UGI endoscopy and 96 (21.1%) awaited repeat testing at the time of analysis. Of the 229 repeated insufficient tests, 184 (80.3%) yielded a satisfactory sample on second test: all patients with a second insufficient test were referred for UGI endoscopy. Of the 608 OCCD tests, 325 (53.5%) demonstrated TFF3 positivity (including those who were also atypia and/or p53 positive). Patients were significantly more likely to demonstrate TFF3 positivity with increasing segment length. TFF3 was positive in 80/282 short segment patients (28.4%); 198/278 long segment patients (71.2%); and 47/48 ultra-long segment patients (97.9%) (*P* < 0.001). Patients with long or ultra-long Barrett's segments were significantly more likely to be high risk (atypia and/or p53 positive) compared to those with short segments (214/326 *versus* 86/282 patients; 65.6% *versus* 30.5%; *P* < 0.001). 43/48 patients (89.6%) with ultra-long Barrett's segments were within the high-risk group.

**Table 1:** OCCD test results according to length of Barrett's segment

OCCD test result	All (*n* = 608)	Short segment (*n* = 282)	Long segment (*n* = 278)	Ultra-long segment (*n* = 48)	*P* *
TFF3 negative	136 (22.4%)	110 (39.0%)	26 (9.4%)	0 (0%)	<0.001
TFF3 positive only	48 (7.9%)	13 (4.6%)	30 (10.8%)	5 (10.4%)	0.020
Atypia only	179 (29.4%)	58 (20.6%)	94 (33.8%)	27 (56.3%)	<0.001
p53 only	24 (3.9%)	7 (2.5%)	15 (5.4%)	2 (4.2%)	0.208
Atypia and p53	97 (16.0%)	21 (7.4%)	62 (22.3%)	14 (29.2%)	<0.001
Insufficient	124 (20.4%)	73 (25.9%)	51 (18.3%)	0 (0%)	<0.001

^*^ *P* as assessed with a 3×2 chi-squared test.

The median time to endoscopy from OCCD test was 2 months (i.q.r. 1–5). *Table 2* summarizes indication for endoscopy. 108/136 patients (79.4%) with a TFF3-negative OCCD test underwent UGI endoscopy to assess suitability for discharge from the surveillance programme. 300/608 endoscopies (49.3%) were performed in response to atypia and/or p53 positivity on OCCD testing (i.e. high-risk group). Seattle protocol biopsies were followed in 150/300 (50.0%) high-risk cases (22/300 had missing data). 27 high-risk patients were not included in this analysis: 12 declined invitation to UGI endoscopy, 8 underwent UGI endoscopy >12 months after OCCD test, 3 had repeat OCCD testing in the first instance, 3 died from unrelated causes, and 1 did not have available biopsy results.

**Table 2:** Indications for UGI endoscopy (*n* = 608)

Indication	*N* (%)
Abnormal OCCD result (atypia ± p53 positive)	300 (49.3%)
Insufficient OCCD result	123 (20.2%)
TFF3 negative OCCD result	108 (17.8%)
Investigation of red flag UGI symptoms	46 (7.6%)
Assessment of ulcer healing detected by OCCD	28 (4.6%)
OCCD sponge detachment	2 (0.3%)
Delayed UGI bleed*	1 (0.2%)

^*^Patient presented with UGI bleed >6 months after OCCD test.

*Table 3* demonstrates endoscopic biopsy result by Barrett's segment length. 50/608 patients (8.2%) had endoscopic biopsies showing HGD, IMC or invasive cancer: this equated to 50/300 (16.7%) within the high-risk group. When extrapolated to the whole Barrett's cohort (*n* = 3745), the prevalence of HGD, IMC or invasive cancer was 1.3%. 88/3745 patients (2.3%) had a normal OCCD test result (i.e. TFF3, atypia and p53 negative) but had endoscopic biopsies demonstrating IM, indefinite for dysplasia or LGD: 22/88 (25.0%) had long-segment Barrett's. No patients had a normal OCCD test but subsequently had endoscopic biopsies demonstrating HGD, IMC or invasive cancer.

**Table 3:** Endoscopic biopsy results according to Barrett's segment length

Endoscopic biopsy result	All (*n* = 608)	Short segment (*n* = 282)	Long segment (*n* = 278)	Ultra-long segment (*n* = 48)	*P* *
No IM	95 (15.6%)	80 (28.4%)	14 (5.0%)	1 (2.1%)	<0.001
Non-dysplastic Barrett's oesophagus	375 (61.7%)	157 (55.7%)	189 (68.0%)	29 (60.4%)	0.011
Indefinite for dysplasia	35 (5.7%)	18 (6.4%)	15 (5.4%)	2 (4.2%)	0.781
LGD	53 (8.7%)	13 (4.6%)	32 (11.5%)	8 (16.6%)	0.002
HGD	26 (4.3%)	9 (3.2%)	13 (4.7%)	4 (8.3%)	0.241
Intramucosal carcinoma	12 (2.0%)	2 (0.7%)	8 (2.9%)	2 (4.2%)	0.095
Adenocarcinoma	11 (1.8%)	3 (1.0%)	6 (2.1%)	2 (4.2%)	0.276
Squamous cell carcinoma	1 (0.2%)	0 (0%)	1 (0.4%)	(0%)	0.552

^*^ *P* as assessed with a 3 × 2 chi-squared test.

*Table 4* demonstrates endoscopic biopsy results by Barrett's surveillance risk group (*n* = 484): patients with an insufficient OCCD test were excluded, as an insufficient result triggered a different clinical decision-making pathway (i.e. repeat OCCD test or routine endoscopy). Of the 50 patients with HGD, IMC or invasive cancer, 46 (92.0%) were high risk (i.e. atypia and/or p53 overexpression on OCCD test), triggering urgent endoscopy. The remaining four patients underwent endoscopy due to insufficient OCCD test (further analysis of endoscopic biopsy results by OCCD test result is available within the Supplementary Material [*Table S2*]). 37/50 (74.0%) patients underwent endoscopic mucosal resection and/or radiofrequency ablation, 7/50 (14.0%) underwent oesophagectomy, 3/50 (6.0%) were treated with radical chemoradiotherapy and 3/50 (6.0%) were palliated due to additional co-morbidities. 2/3745 Barrett's surveillance patients (0.1%) died following a diagnosis of OAC.

**Table 4:** Endoscopic biopsy results compared with Barrett's surveillance risk group (*n* = 484)

	All patients (*n* = 484)	Ultra-low risk (+*n* = 126)	Low risk (*n* = 37)	Moderate risk (*n* = 21)	High risk (*n* = 300)
No IM	67 (13.8%)	48 (71.6%)	5 (7.5%)	3 (4.5%)	11 (16.4%)
Non dysplastic Barrett's oesophagus	290 (59.9%)	70 (24.1%)	31 (10.7%)	16 (5.5%)	173 (59.7%)
Indefinite for dysplasia	33 (6.8%)	5 (15.2%)	0 (0%)	1 (3.0%)	27 (81.8%)
LGD	48 (9.9%)	3 (6.3%)	1 (2.1%)	1 (2.1%)	43 (89.6%)
HGD	23 (4.8%)	0 (0%)	0 (0%)	0 (0%)	23 (100%)
Intramucosal carcinoma	12 (2.5%)	0 (0%)	0 (0%)	0 (0%)	12 (100%)
Adenocarcinoma	10 (2.1%)	0 (0%)	0 (0%)	0 (0%)	10 (100%)
Squamous cell carcinoma	1 (0.2%)	0 (0%)	0 (0%)	0 (0%)	1 (100%)

## Discussion

The present study is the first in clinical practice across a wide geographical location to demonstrate the diagnostic accuracy of OCCD testing and feasibility of its ongoing use as a diagnostic triage tool to UGI endoscopy within the Barrett's surveillance cohort.

Previous literature demonstrates incidence rates of 8%, 4% and 1–2% of LGD, HGD and OAC in patients undergoing endoscopic Barrett's surveillance respectively^18,19^. Our initial results present similar overall incidence rates at endoscopic biopsy, implying OCCD testing is successfully identifying those patients requiring urgent UGI endoscopy, although longer-term endoscopic data are required to validate this claim.

Prior to the COVID-19 pandemic, all Barrett's patients underwent endoscopic surveillance, whereas only 16.2% of our cohort required endoscopy within 12 months of OCCD testing. Estimates suggest the CytoSCOT programme has saved NHS Scotland in excess of £200,000 for Barrett's surveillance since its inception in September 2020^20^, while safely identifying those at increased cancer risk. These financial savings are expected to multiply with the continued expansion of OCCD testing.

Within the HGD, IMC or invasive cancer cohort, 92.0% had an abnormal OCCD test result (atypia and/or p53 overexpression), triggering urgent referral for UGI endoscopy: this increased to 100% once insufficient tests were removed. Previous trial data have supported the use of an OCCD biomarker panel in conjunction with clinical details to generate a Barrett's risk group, thereby enabling identification of patients at increased risk of dysplasia or malignancy^11,17^. Our real-world results support this and demonstrate Barrett's risk group is a useful predictor of dysplasia or cancer at endoscopic biopsy, which could aid prioritization of limited endoscopy resources. The present data suggest OCCD testing has a positive predictive value (PPV) of 16.7% for HGD, IMC or invasive cancer, compared to 30.8% in previously published literature^11^: this may be due to the lower adherence to Seattle protocol biopsies within our cohort, and reflects the real-world nature of our retrospective data set. This highlights the importance of stringent follow-up of the remaining 83.3% of the high-risk group: this cohort is likely to benefit from more frequent surveillance than the current recommended intervals by the BSG^2^.

Increasing segment length is an independent risk factor for the development of dysplasia^21^. LGD was significantly more common in those with longer Barrett's segments, but not HGD or cancer. Patients with longer segments were significantly more likely to demonstrate atypia and/or p53 overexpression on OCCD testing, again suggesting that these patients may benefit from more frequent surveillance to detect dysplastic change.

Notably, the 608 patients included within the analysis are a higher-risk group compared to the overall Barrett's surveillance population: 300/608 (49.3%) demonstrated positive OCCD biomarkers for atypia and/or p53. This higher proportion of high-risk patients is further compounded by the negative impact of delayed surveillance on endoscopic pathology patterns due to the COVID-19 pandemic^16^. Within our described cohort undergoing UGI endoscopy, only 48/608 patients (7.9%) had an OCCD test demonstrating TFF3 positivity alone. Most patients with a TFF3-positive only result within the Barrett's cohort were pushed to their next surveillance interval as per BSG guidelines^2^: they did not undergo UGI endoscopy and were not included in this analysis. This also explains the higher proportion of insufficient (20.4%) and TFF3-negative (22.4%) results within this cohort compared to the wider Barrett's group, as repeat insufficient tests or a TFF3-negative result may trigger referral for endoscopy. Where the OCCD result was insufficient for analysis, repeat OCCD testing was recommended in the first instance, although the decision to proceed directly to UGI endoscopy was left at clinicians’ discretion. In TFF3-negative cases, there was clinical variability on decision to continue surveillance or offer endoscopy to clarify safe discharge. Notably, patients were only discharged from surveillance if IM was not identified on endoscopic biopsies.

Of the 608 patients, 22.4% had a TFF3-negative result, including 9.4% of patients with long-segment Barrett's. This may be due to sampling error, as well as the inclusion of cases without true Barrett's oesophagus (for example, those with an irregular Z-line) and short segments <1 cm in our data set. This is supported by the fact that the proportion of TFF3-positive tests increased with segment length. Repeat OCCD testing was recommended in the first instance for longer Barrett's segments to clarify if the result was spurious. The data set also highlights a small proportion of patients (88/3745, 2.3%) within the TFF3-negative group who had endoscopic biopsies confirming either IM or LGD (i.e. ‘missed’ pathology on OCCD testing). This is complicated by the fact that patients with a TFF3-negative OCCD test did not all routinely undergo UGI endoscopy. The aim of OCCD testing is to risk stratify to reduce burden on patients and endoscopy services while not missing cancer diagnoses: previous literature has suggested HGD may be detected in <2% in the low-risk group and approximately 8% in the moderate-risk group^11^. Although UGI endoscopy remains the gold standard for Barrett's surveillance, endoscopic biopsies are subject to significant sampling bias and may be fraught with user error. Post-endoscopy incident oesophageal cancer is estimated to account for 14% of the oesophageal cancer burden^22^, with published missed OAC rates in the Barrett's surveillance population within 1 year of index endoscopy of 23–30%^6–8^. Thus, neither UGI endoscopy nor the OCCD is the perfect test, with both investigations posing the risk of missed pathology. Reassuringly, there were no cases of missed cancers within 1 year of OCCD testing within our cohort; however, more follow-up data are required in the low- and moderate-risk groups, as well as those in the high-risk group with no evidence of dysplasia within endoscopic biopsies, before definitive conclusions can be drawn.

This study has several limitations. Forward referral for endoscopy was not standardized nationally, with this decision left at clinicians’ discretion. This resulted in significant heterogeneity in decision-making pathways between local health boards, reflecting the real-world nature of this work and the requirement for pragmatism when interpreting its results. Development of national guidelines for appropriate surveillance methods will be important as follow-up data emerge. In the present study, the median follow-up time is currently only 14 months. However, previous literature suggests that progression to dysplasia or malignancy usually occurs early within the Barrett's surveillance population^18^: continued follow-up of our cohort will be required to ensure no cancer diagnoses are missed. We are also unable to draw definite conclusions as to whether future follow-up should be with OCCD testing *versus* endoscopy at this juncture. Although it may be hypothesized that OCCD testing could replace endoscopy as the first-line investigation for Barrett's surveillance, analysis of patients undergoing repeat OCCD testing at their next surveillance interval will be critical to formalize this pathway long term. Furthermore, endoscopic biopsy results and length of surveillance period prior to the CytoSCOT programme were not accessible on a national level to enable accurate comparison between the two Barrett's surveillance modalities. Finally, there was no access to national data for the few patients who proceeded direct to endoscopic surveillance, either due to contraindications to OCCD testing or inability to swallow the OCCD: this number is hypothesized to be negligible compared to the proportion who underwent OCCD testing, as the latter became the standard of care for Barrett's surveillance during the study period across Scotland.

Within the Scottish real-world setting, OCCD testing demonstrates sound diagnostic accuracy as a triage tool in highlighting high-risk Barrett's surveillance patients requiring urgent UGI endoscopy. Moving forward, OCCD testing should have a standardized role in clinical practice to support overwhelmed endoscopy and histopathology services within the Barrett's population. While urgent investigation is recommended for high-risk patients, further data are required to establish definitive future surveillance guidelines.

## Supplementary Material

znae117_Supplementary_Data

## Data Availability

Data will be made available upon reasonable request to the corresponding author. This study was not pre-registered in an independent institutional registry as it was performed using retrospective cohort data with appropriate ethical approval.

## References

[1] Domper Arnal MJ, Ferrández Arenas Á, Lanas Arbeloa Á. Esophageal cancer: risk factors, screening and endoscopic treatment in Western and Eastern countries. World J Gastroenterol 2015;21:7933–7943 26185366 10.3748/wjg.v21.i26.7933 PMC4499337

[2] Fitzgerald RC, di Pietro M, Ragunath K, Ang Y, Kang J-Y, Watson P et al British Society of Gastroenterology guidelines on the diagnosis and management of Barrett’s oesophagus. Gut 2014;63:7–42 24165758 10.1136/gutjnl-2013-305372

[3] Desai TK, Krishnan K, Samala N, Singh J, Cluley J, Perla S et al The incidence of oesophageal adenocarcinoma in non-dysplastic Barrett’s oesophagus: a meta-analysis. Gut 2012;61:970–976 21997553 10.1136/gutjnl-2011-300730

[4] Shaheen NJ, Overholt BF, Sampliner RE, Wolfsen HC, Wang KK, Fleischer DE et al Durability of radiofrequency ablation in Barrett’s esophagus with dysplasia. Gastroenterology 2011;141:460–468 21679712 10.1053/j.gastro.2011.04.061 PMC3152658

[5] El-Serag HB, Naik AD, Duan Z, Shakhatreh M, Helm A, Pathak A et al Surveillance endoscopy is associated with improved outcomes of oesophageal adenocarcinoma detected in patients with Barrett’s oesophagus. Gut 2016;65:1252–1260 26311716 10.1136/gutjnl-2014-308865

[6] Visrodia K, Singh S, Krishnamoorthi R, Ahlquist DA, Wang KK, Iyer PG et al Magnitude of missed esophageal adenocarcinoma after Barrett’s esophagus diagnosis: a systematic review and meta-analysis. Gastroenterology 2016;150:599–607.e7; quiz e14–5 26619962 10.1053/j.gastro.2015.11.040 PMC4919075

[7] van Putten M, Johnston BT, Murray LJ, Gavin AT, McManus DT, Bhat S et al ‘Missed’ oesophageal adenocarcinoma and high-grade dysplasia in Barrett’s oesophagus patients: a large population-based study. United European Gastroenterol J 2018;6:519–528 10.1177/2050640617737466 PMC5987274 29881607

[8] Wani S, Holmberg D, Santoni G, Kauppila JH, Farkkila M, von Euler-Chelpin M et al Magnitude and time-trends of post-endoscopy esophageal adenocarcinoma and post-endoscopy esophageal neoplasia in a population-based cohort study: the Nordic Barrett’s esophagus study. Gastroenterology 2023;165:909–919 37279832 10.1053/j.gastro.2023.05.044

[9] Edwards C, Penman ID, Coleman M. Gastrointestinal endoscopy during COVID-19: when less is more. Frontline Gastroenterol 2020;11:256–257 32582419 10.1136/flgastro-2020-101492 PMC7307043

[10] Fitzgerald RC, di Pietro M, O’Donovan M, Maroni R, Muldrew B, Debiram-Beecham I et al Cytosponge-trefoil factor 3 *versus* usual care to identify Barrett’s oesophagus in a primary care setting: a multicentre, pragmatic, randomised controlled trial. Lancet 2020;396:333–344 32738955 10.1016/S0140-6736(20)31099-0 PMC7408501

[11] Pilonis ND, Killcoyne S, Tan WK, O’Donovan M, Malhotra S, Tripathi M et al Use of a cytosponge biomarker panel to prioritise endoscopic Barrett’s oesophagus surveillance: a cross-sectional study followed by a real-world prospective pilot. Lancet Oncol 2022;23:270–278 35030332 10.1016/S1470-2045(21)00667-7 PMC8803607

[12] Ross-Innes CS, Chettouh H, Achilleos A, Galeano-Dalmau N, Debiram-Beecham I, MacRae S et al Risk stratification of Barrett’s oesophagus using a non-endoscopic sampling method coupled with a biomarker panel: a cohort study. Lancet Gastroenterol Hepatol 2017;2:23–31 28404010 10.1016/S2468-1253(16)30118-2

[13] Ross-Innes CS, Debiram-Beecham I, O’Donovan M, Walker E, Varghese S, Lao-Sirieix P et al Evaluation of a minimally invasive cell sampling device coupled with assessment of trefoil factor 3 expression for diagnosing Barrett’s esophagus: a multi-center case–control study. PLoS Med 2015;12:e1001780 25634542 10.1371/journal.pmed.1001780 PMC4310596

[14] Maroni R, Barnes J, Offman J, Scheibl F, Smith SG, Debiram-Beecham I et al Patient-reported experiences and views on the Cytosponge test: a mixed-methods analysis from the BEST3 trial. BMJ Open 2022;12:e054258 10.1136/bmjopen-2021-054258 PMC8990713 35393308

[15] Scottish Government . Endoscopy and Urology Diagnostic: Recovery and Renewal Plan. Scotland: Scottish Government, 2021 [updated 2021; cited 2023 September 25]. https://www.gov.scot/publications/endoscopy-urology-diagnostic-recovery-renewal-plan/pages/1

[16] Chien S, Glen P, Penman I, Bryce G, Cruickshank N, Miller M et al National adoption of an esophageal cell collection device for Barrett’s esophagus surveillance: impact on delay to investigation and pathological findings. Dis Esophagus 2024;37: doae002 10.1093/dote/doae002 38267082

[17] Landy R, Killcoyne S, Tang C, Juniat S, O’Donovan M, Goel N et al Real-world implementation of non-endoscopic triage testing for Barrett’s oesophagus during COVID-19. QJM 2023;116:659–666 37220898 10.1093/qjmed/hcad093 PMC10497181

[18] Peters Y, Honing J, Kievit W, Kestens C, Pestman W, Nagtegaal ID et al Incidence of progression of persistent nondysplastic Barrett’s esophagus to malignancy. Clin Gastroenterol Hepatol 2019;17:869–877.e5 30213587 10.1016/j.cgh.2018.08.033

[19] de Jonge PJ, van Blankenstein M, Looman CW, Casparie MK, Meijer GA, Kuipers EJ. Risk of malignant progression in patients with Barrett’s oesophagus: a Dutch nationwide cohort study. Gut 2010;59:1030–1036 20639249 10.1136/gut.2009.176701

[20] National Institute for Health and Care Excellence . Cytosponge for Detecting Abnormal Cells in the Oesophagus. National Institute for Health and Care Excellence, 2020. https://www.nice.org.uk/advice/mib240/chapter/Summary (accessed 11 October 2023)

[21] Anaparthy R, Gaddam S, Kanakadandi V, Alsop BR, Gupta N, Higbee AD et al Association between length of Barrett’s esophagus and risk of high-grade dysplasia or adenocarcinoma in patients without dysplasia. Clin Gastroenterol Hepatol 2013;11:1430–1436 23707463 10.1016/j.cgh.2013.05.007

[22] Vajravelu RK, Kolb JM, Thanawala SU, Scott FI, Han S, Singal AG et al Characterization of prevalent, post-endoscopy, and incident esophageal cancer in the United States: a large retrospective cohort study. Clin Gastroenterol Hepatol 2022;20:1739–1747 33549872 10.1016/j.cgh.2021.02.005 PMC8895727

