# Supplementary material for: Oesophageal cell collection device and biomarker testing to identify high-risk Barrett's patients requiring endoscopic investigation
Source: Br J Surg. 2024 May 13;111(5):znae117. doi: 10.1093/bjs/znae117 (PMC11089076; doi:10.1093/bjs/znae117)
Supplement: znae117_Supplementary_Data [file znae117_supplementary_data.docx]

**Title**

Oesophageal cell collection device and biomarker testing to identify high-risk Barrett’s patients requiring endoscopic investigation

**Authors**:

Siobhan Chien^1,2^, Paul Glen^3^, Ian Penman^4^, Neil Cruickshank^5^, Gavin Bryce^6^, Andrew Crumley^7^, Perminder Phull^8^, Michael Miller^9^, Jonathan Fletcher^10^, Ivan Gunjaca^11^, Jeyakumar Apollos^12^, Kevin Robertson^13^, Grant Fullarton^2^ (on behalf of the CytoSCOT group)

**Author’s affiliation**:

^1^ School of Cancer Sciences, University of Glasgow, Garscube Estate, Switchback Road, Bearsden, Glasgow, UK, G61 1QH.

^2^ Centre for Sustainable Delivery, Golden Jubilee National Hospital, Clydebank, Glasgow, UK, G81 4DN.

^3^ Department of General Surgery, Queen Elizabeth University Hospital, Glasgow, UK, G51 4TF.

^4^ Centre for Liver & Digestive Disorders, Royal Infirmary of Edinburgh, Edinburgh, UK, EH16 4SA.

^5^ Department of General Surgery, Victoria Hospital, Kirkcaldy, UK, KY2 5AH.

^6^ Department of General Surgery, University Hospital Wishaw, Wishaw, UK, ML2 0DP.

^7^ Department of General Surgery, Forth Valley Royal Hospital, Larbert, UK, FK5 4WR.

^8^ Department of Gastroenterology, Aberdeen Royal Infirmary, Aberdeen, UK, AB25 2ZN.

^9^ Department of Gastroenterology, Ninewells Hospital, Dundee, UK, DD2 1SG.

^10^ Department of Gastroenterology, Borders General Hospital, Melrose, UK, TD6 9BS.

^11^ Department of Gastroenterology, Raigmore Hospital, Inverness, UK, IV2 3UJ.

^12^ Department of General Surgery, Dumfries & Galloway Royal Infirmary, Dumfries, UK, DG2 8RX.

^13^ Department of General Surgery, University Hospital Crosshouse, Kilmarnock, UK, KA2 0BE.

**Corresponding Author**:

Siobhan Chien

Centre for Sustainable Delivery, Golden Jubilee National Hospital, Clydebank, Glasgow, UK, G81 4DN.

siobhan.chien6@gjnh.scot.nhs.uk

ORCID ID: 0000-0001-9657-193X

**Supplementary Materials - Index**

| **Supplementary Methods** |  |
| --- | --- |
| N/A | *pag. 2* |
| **Supplementary Results** |  |
| N/A | *pag. 3* |
| **Supplementary Appendixes** |  |
| N/A | *pag. 4* |
| **Supplementary Figures and Tables** |  |
| Figure S1 | *pag. 6* |
| Figure S2 | *pag. 7* |
| Table S1 | *pag. 8* |
| Table S2 | *pag. 9* |
|  |  |
| **References** | *pag. 10* |
|  |  |

**Supplementary Methods**

**N/A**

**Supplementary Results**

**N/A**

**Supplementary Appendixes**

**N/A**

**Supplementary Figures and Tables**

**Figure S1:** OCCD test decision tree based on risk group and biomarker status, as published by Landy *et al* (1).

**Figure S2**: Inclusion criteria for data analysis.

**Table S1:** OCCD test results in the whole Barrett’s surveillance cohort (n=4204).

| **OCCD test result** | **N (%)** |
| --- | --- |
| TFF3 negative | 1451 (34.5%) |
| TFF3 positive only | 1970 (46.8%) |
| Atypia only | 191 (4.5%) |
| p53 only | 30 (0.7%) |
| Atypia *and* p53 positive | 106 (2.5%) |
| Insufficient | 456 (10.8%) |

Table S2: Endoscopic biopsy results compared to OCCD test result (n=608).

| **Endoscopic biopsy result** | **All (n=608)** | **OCCD test result** | | | | | |
| --- | --- | --- | --- | --- | --- | --- | --- |
|  |  | **TFF3 negative (n=136)** | **TFF3 positive only (n=48)** | **Atypia only (n=179)** | **P53 only (n=24)** | **Atypia AND p53 (n=97)** | **Insufficient (n=124)** |
| No IM | 95 (15.6%) | 48 (35.3%) | 8 (16.7%) | 11 (6.1%) | 0 (0%) | 0 (0%) | 28 (22.6%) |
| Non-dysplastic Barrett’s oesophagus | 375 (61.7%) | 80 (58.8%) | 37 (77.1%) | 121 (67.6%) | 17 (70.8%) | 35 (36.1%) | 85 (68.5%) |
| Indefinite for dysplasia | 35 (5.8%) | 5 (3.7%) | 1 (2.1%) | 15 (8.4%) | 1 (4.2%) | 11 (11.3%) | 2 (1.6%) |
| LGD | 53 (8.7%) | 3 (2.2%) | 2 (4.2%) | 20 (11.2%) | 5 (20.8%) | 18 (18.6%) | 5 (4.0%) |
| HGD | 26 (4.3%) | 0 (0%) | 0 (0%) | 6 (3.4%) | 0 (0%) | 17 (17.5%) | 3 (2.4%) |
| Intramucosal carcinoma | 12 (2.0%) | 0 (0%) | 0 (0%) | 2 (1.1%) | 1 (4.2%) | 9 (9.3%) | 0 (0%) |
| Adenocarcinoma | 11 (1.8%) | 0 (0%) | 0 (0%) | 3 (1.7%) | 0 (0%) | 7 (7.2%) | 1 (0.8%) |
| Squamous cell carcinoma | 1 (0.2%) | 0 (0%) | 0 (0%) | 1 (0.6%) | 0 (0%) | 0 (0%) | 0 (0%) |

**References**

1. Landy R, Killcoyne S, Tang C, Juniat S, O'Donovan M, Goel N, et al. Real-world implementation of non-endoscopic triage testing for Barrett's oesophagus during COVID-19. Qjm. 2023;116(8):659-66.
